# Less experienced observers assess piglet castration-induced acute pain differently than experienced observers: A pilot study

**DOI:** 10.1371/journal.pone.0309684

**Published:** 2024-09-04

**Authors:** Gustavo Venâncio da Silva, Magdiel Lopez-Soriano, Monique Danielle Pairis-Garcia, Pedro Henrique Esteves Trindade

**Affiliations:** 1 Laboratory of Applied Artificial Intelligence on Health, Department of Anesthesiology, Botucatu Medical School, São Paulo State University (Unesp), Botucatu, São Paulo, Brazil; 2 Extension Department, University of Missouri, Columbia, Missouri, United States of America; 3 Global Production Animal Welfare Laboratory, Department of Population Health and Pathobiology, College of Veterinary Medicine, North Carolina State University (NCSU), Raleigh, North Carolina, United States of America; University of Life Sciences in Lublin, POLAND

## Abstract

Behavioral pain scales have been helpful for standardized swine pain assessment. However, it is still unknown if observers’ experience influences the scale score. We conducted a pilot study to investigate how three different levels of swine experience influenced how observers scored castration pain in piglets using Unesp-Botucatu Pig Composite Acute Pain Scale (UPAPS). We used a database from UPAPS scores from pigs undergoing surgical castration in a previous study. Scores were attributed by six observers with Little to no experience (n = 2), Some experience (n = 2) and Extensive experience (n = 2). Reliability was estimated using the intraclass correlation coefficient, agreement was investigated by Bland-Altman analysis, predictive capacity was estimated using the area under the curve (AUC), and statistical differences were tested using a regression model. We found that intra-experience levels reliability were satisfactory (Little to no: 0.72, Some: 0.81, Extensive: 0.84), but inter-experience reliability was lower (0.42). Little to no experience observers had poor agreement with other observers, with a bias toward underscoring UPAPS (bias of 0.94 vs. Some, 1.17 vs. Extensive). Predictive capacity was similar between all observers (AUC, Little to no: 71.94%, Some: 76.10%, Extensive: 79.09%, p > 0.05). Regression model confirmed underscoring of Little to no experience observers (mean ± standard error; Little to no: 1.09 ± 0.14; Some: 2.02 ± 0.23; Extensive: 2.25 ± 0.22; p < 0.05). We concluded that minimal experience, as Some experience observers have in the swine industry, is sufficient for them to score UPAPS in a similar way than more experienced observers. The present pilot study supports the enhancement and implementation of UPAPS on farm and laboratory settings by minimally qualified observers, improving swine welfare in the short and long term.

## Introduction

Pain perception is influenced by social, cultural and individual factors, therefore, pain diagnosis in non-human animals may be challenging [1]. Behavioral pain scales have been successfully implemented to reduce variability and improve objectivity in pain diagnosis, as well as promote good animal welfare in farm and laboratory settings [2]. Whether raised for pork production [3] or scientific research [4], pigs undergo painful procedures such as tail docking, teeth resection, surgical castration, ear tagging and notching [5–7]. These procedures result in acute pain experienced by pigs, which compromises individual animal welfare and puts into question the ethical obligation to minimize pain and suffering experienced by animals in our care and responsibility [8].

The Unesp-Botucatu Pig Composite Acute Pain Scale (UPAPS) was developed for diagnosing pain in weaned and pre-weaned piglets undergoing surgical castration [9, 10]. Since its development, UPAPS has been used to diagnose pain and evaluate analgesic drug efficacy [11, 12] and was considered the only scale with high validation evidence to assess acute pain in swine [2]. Although of great importance, all behavioral scales, including the UPAPS, are susceptible to some subjectivity due to observers’ individual experiences [13]. The UPAPS was initially developed and validated by senior researchers with extensive experience in pain assessment, however, in a real-world scenario, pig pain will likely be diagnosed by caretakers or producers with limited to no formal pain assessment training and experience. This phenomenon has been documented in previous work assessing pain in cats using observers with differing backgrounds [13] documenting differences between observers. Scale design, observers’ responding strategies, or familiarity with the species may all be factors in why observers assess pain differently [14]. Gaining insights into these differences from the swine perspective is important not only to ensure pain in pigs is appropriately assessed, but also refining or reducing UPAPS complexity is necessary for different experience levels. However, to date there are no publications addressing how UPAPS scores would differ from observers with little experience compared with the more experienced ones. Hence, the objective of this pilot study was to investigate if three different levels of swine experience (no experience, some, extensive) can influence observers to score surgical castration pain in piglets using UPAPS. We found that observers with less than three months of experience in the swine industry underscore pain in piglets.

## Methods

The present study was approved by the Institutional Animal Care and Use Committee of North Carolina State University (#20–113). All piglets were handled following the Guide for the Care and Use of Agricultural Animal in Research and Teaching [15] and all piglets undergoing castration received pain management before the procedure. Data collected for this study was part of a larger project [12], thus contributing to the four R’s of animal experimentation [16, 17]. Reporting is in accordance with the ARRIVE guidelines [18].

### Animals, housing conditions and surgical castration

A total of 29 Large White x Duroc cross male piglets (15 litters, approximately 2 piglets per litter) were enrolled in the study. Housing conditions and complete surgical castration procedure description can be found in the previously published study [12].

### Pain assessment

Whole-body behavior was filmed continuously for 4 min at three timepoints. Timepoints were chosen to account for three levels of pain severity expressed as previously described [9, 10]. Timepoints included: 1 h before castration (pain-free), immediately after castration (severe pain), and 3 h post-castration (mild to moderate pain) [9–12]. Video records were obtained using a high-definition camera (Sony HDR-CX405®; New York, NY, USA) placed on a tripod approximately 30 cm from the crate at a 122-cm height. A total of 177 videos were obtained (29 per timepoint), 348 min in total, without editing video clips.

The UPAPS is composed of multiple behavioral responses used to diagnose and quantify pain. The UPAPS was previously validated in piglets [10] using five behavioral items specific to posture, interaction and interest in the surroundings, activity, attention to the affected area and miscellaneous behaviors. These behavioral items are descriptive and composed of four score levels: ‘0’ (normal behavior), and ‘1’, ‘2’ or ‘3’, according to the presence of pain-related behaviors (Table 1). Individual items were scored and total pain scores were calculated by summing the five behavioral items into one total score per piglet by timepoint (0–15).

**Table 1:** Description and tutorial videos for each behavioral response of the Unesp-Botucatu Pig Composite Pain Scale (UPAPS) system [9, 10].

Item	Score	Score/criterion	Links to videos
Posture	0	Normal (any position, apparent comfort, relaxed muscles) or sleeping	https://youtu.be/QSosCD2SD4E
	1	Changes posture, with discomfort	https://youtu.be/SpaWsFCrPxE
	2	Changes posture, with discomfort, and protects the affected area	https://youtu.be/VjSlsRrG8yA
	3	Quiet, tense, and back arched	https://youtu.be/pm4hJ5163ao
Interaction and interest in the surroundings	0	Interacts with other animals; interested in the surroundings or sleeping	https://youtu.be/-880STgYq2I
	1	Only interacts if stimulated by other animals; interested in the surroundings.	https://youtu.be/nXjOdwn3dyw
	2	Occasionally moves away from the other animals, but accepts approaches; shows little interest in the surroundings	https://youtu.be/2k2JDr5U6As
	3	Moves or runs away from other animals and does not allow approaches; disinterested in the surroundings	https://youtu.be/se70oYXcWFw
Activity	0	Moves normally or sleeping	https://youtu.be/cC75t7L5-YA
	1	Moves with less frequency	https://youtu.be/lQo9wq8LAn8
	2	Moves constantly, restless	https://youtu.be/YQRJjijLvpk
	3	Reluctant to move or does not move	https://youtu.be/Zyx0G3Wpt8o
Attention to the affected area		A. Elevates pelvic limb or alternates the support of the pelvic limb	https://youtu.be/UD99ftO7HE0
		B. Scratches or rubs the painful area	https://youtu.be/7idfFk1harE
		C. Moves and/or runs away and/or jumps after injury of the affected area	https://youtu.be/u-Pqubom278
		D. Sits with difficulty	https://youtu.be/ETNEOCVV4h0
	0	All the above behaviors are absent	
	1	Presence of one of the above behaviors	
	2	Presence of two of the above behaviors	
	3	Presence of three or all the above behaviors	
Miscellaneous behaviors		A. Wags tail continuously and intensely	https://youtu.be/pU5dGZFNRHc
		B. Bites the bars or objects	https://youtu.be/cF3dsq7gMtk
		C. The head is below the line of the spinal column.	https://youtu.be/ZcIgngclRpI
		D. Presents difficulty in overcoming obstacles (example: another animal)	https://youtu.be/HlvdOI3lGuY
	0	All the above behaviors are absent	
	1	Presence of one of the above behaviors	
	2	Presence of two of the above behaviors	
	3	Presence of three or all the above behaviors	

### Observers experience

Pain assessment was performed by six observers with three levels of experience, classified as (*i*) Little to no experience: observers with almost no work on farm or research experience with pigs (n = 2); (*ii*) Some experience: observers with less than one year of farm or research experience with pigs (n = 2); and (*iii*) Extensive experience: observers with more than a year of farm or research experience with pigs (n = 2). Little to no experience and Some experience observers were recruited through a veterinary summer scholars research program at the North Carolina State University. All observers underwent training for scoring on the UPAPS, provided by one of the observers with Extensive experience. During training, the trainer provided video examples, definitions, and descriptions of each behavior, and trainees were required to score 20 masked videos of piglets in painful and pain free states (10 videos of each).

### Statistical analysis

Data were analyzed using R software within the integrated RStudio environment [19] (Version 4.2.2; RStudio, Inc., Boston, MA, USA). Functions and packages used were presented in the format ’package::function’ corresponding to the computer programming language in R. A significance of 5% was considered for all inference tests. A palette of colors distinguishable by people with common forms of color blindness was used in all figures (ggplot2::scale_colour_viridis_d).

Intraclass correlation coefficient (ICC), two-way random effects model, type agreement multiple observers, and its 95% confidence interval (CI) (irr::icc) were used to evaluate the inter-observer, inter-group and intra-group reliability of the UPAPS total sum for experience level groups (Little to no experience, Some experience, and Extensive experience). The interpretation of ICC was as follows: very good: 0.81–1.0; good: 0.61–0.80; moderate: 0.41–0.60; reasonable: 0.21–0.4; and poor ≤ 0.2 [20].

Bland-Altman test for repeated measures [21] and Lin’s concordance correlation coefficient (CCC) [22] (SimplyAgree::agree_reps) were used to verify the agreement of UPAPS total sum assessed by each experience level group, as proposed previously [23]. As there were three groups and Bland-Altman analysis only allows pairwise comparisons, it was required three analyses to evaluate the agreement between all three possible combinations. Bland-Altman analysis is suitable for detecting bias referring to the difference between two experience level groups [21]. In addition, the Bland-Altman analysis provides the limit of agreement (LoA), which indicates the expected range where most differences between experience level groups should occur [21]. A simple linear regression (stats::lm) was conducted to analyze the proportional bias between experience levels [24]. Proportional bias represents an increase in the difference between experience level groups evaluated at higher or lower UPAPS total sum. The difference in the UPAPS total sum between two experience level groups was used as a response variable, and the average of the UPAPS total sum between the two groups was used as an explanatory variable. Three simple linear regressions were required to cover for the three possible combinations between the three experience level groups. The heteroskedasticity of the linear model was tested with the Breusch Pagan test (olsrr::ols_test_breusch_pagan).

Multilevel negative binomial modeling (lme4::glmer.nb) was employed to investigate the influence of timepoint and experience level (explanatory variables) on UPAPS total score (response variable). The best combination of fixed and random effects were identified according to the lowest Bayesian information criterion (stats::BIC) using preliminary models. Best-fit preliminary model did not consider the interaction between predictive variables, in other words, the dynamic of the UPAPS total sum for each experience level group was same for all timepoints. Piglets nested within each litter were considered as random effects. Bonferroni correction was used for adjusting the multiple comparisons in the post-hoc test (lsmeans::lsmeans and multcomp::cld).

Multilevel binomial modeling (lme4::glmer) was employed to investigate the influence of experience level group (explanatory variable) on UPAPS behaviors as dummy variables (binomial response variable). Dummy variables were created using fastDummies::dummy_columns for each score level. For example: Posture item was transformed into four items: Posture 0, Posture 1, Posture 2 and Posture 3, and each one of them was a binary variable of ‘0’, for when the score is not given, and ‘1’, for when the score is given. Bonferroni correction was also used for adjusting the multiple comparisons in the post-hoc test (lsmeans::lsmeans and multcomp::cld).

Receiver operating characteristic (ROC) curve was fitted to investigate experience level groups discriminative capacities. Response variable was Condition, a new binary variable that assumed ‘0’ when the assessment was performed in a video recorded at 1 h before castration (no pain) and ‘1’ when the assessment was performed in a video recorded immediately after castration (severe pain), similar as conducted previously [10, 25]. The 3 h post-castration timepoint was not considered in this analysis. Explanatory variable was UPAPS total sum. ROC curves were then used to estimate area under the curve (AUC), sensitivity and specificity. AUC were compared statistically using DeLong test.

## Results

### Reliability

**Fig 1:**
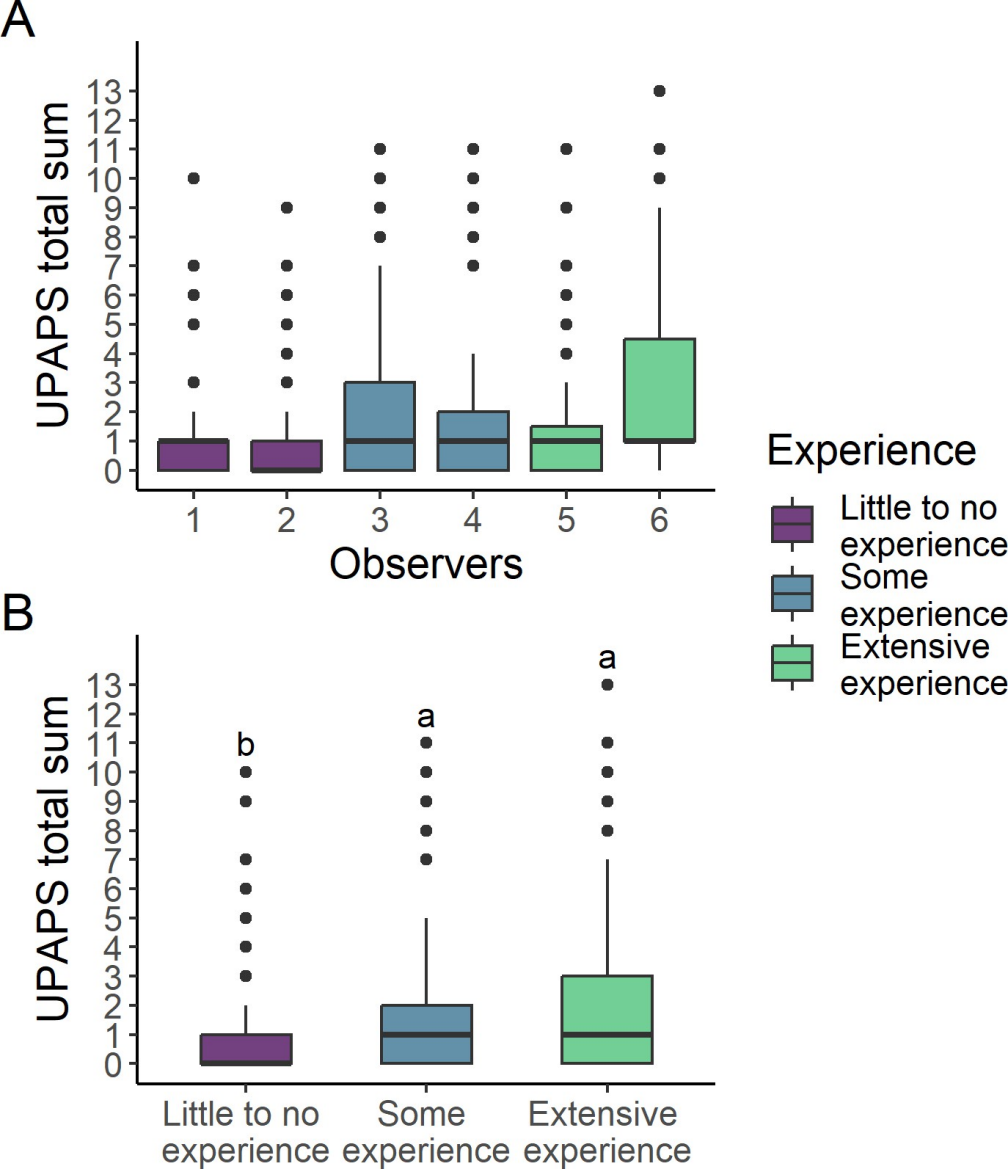
Observers scoring on UPAPS total sum. **(A)** Overall observers scoring on UPAPS total sum boxplot across experience levels. **(B)** UPAPS total score by different levels of experience: little to no experience, some experience and extensive experience. (Letters indicate statistical differences (p < 0.05) found in the Bonferroni post-hoc test (a>b). UPAPS: Unesp-Botucatu Pig Composite Acute Pain Scale).

Overall inter-observer reliability was “very good”, intra-experience level reliability was “good” and “very good” and inter-experience level reliability was “moderate” (Table 2). Score distribution of each observer is presented in Fig 1A.

**Table 2:** Observers’ reliability on scoring UPAPS total sum.

Reliability	ICC	p-value
Overall inter-observer	**0.91** (0.88–0.94)	< 0.001
Inter-experience level	0.42 (0.25–0.55)	< 0.001
Intra-extensive experience	**0.84** (0.75–0.89)	< 0.001
Intra-some experience	**0.81** (0.70–0.87)	< 0.001
Intra-little to no experience	**0.72** (0.57–0.82)	< 0.001

UPAPS: Unesp-Botucatu pig composite acute pain scale; ICC: Intraclass correlation coefficient; Interpretation of reliability: very good 0.81–1.0; good 0.61–0.80; moderate 0.41–0.60; reasonable 0.21–0.40; poor < 0.20. Bold type corresponds to values ≥ 0.61 [14].

### UPAPS total sum modeling

Parameters of the negative binomial modeling are presented in S1 Table. In the Bonferroni post-hoc test, Little to no experience level assessed a lower UPAPS total sum than Some experience and Extensive experience level (Fig 1B). Post-hoc analysis also confirmed that a higher UPAPS total sum was present immediately post-castration, followed by 3 h post-castration and lower values 1 h before castration considering all observer levels (S1 Fig).

### Bland-Altman analysis

**Fig 2:**
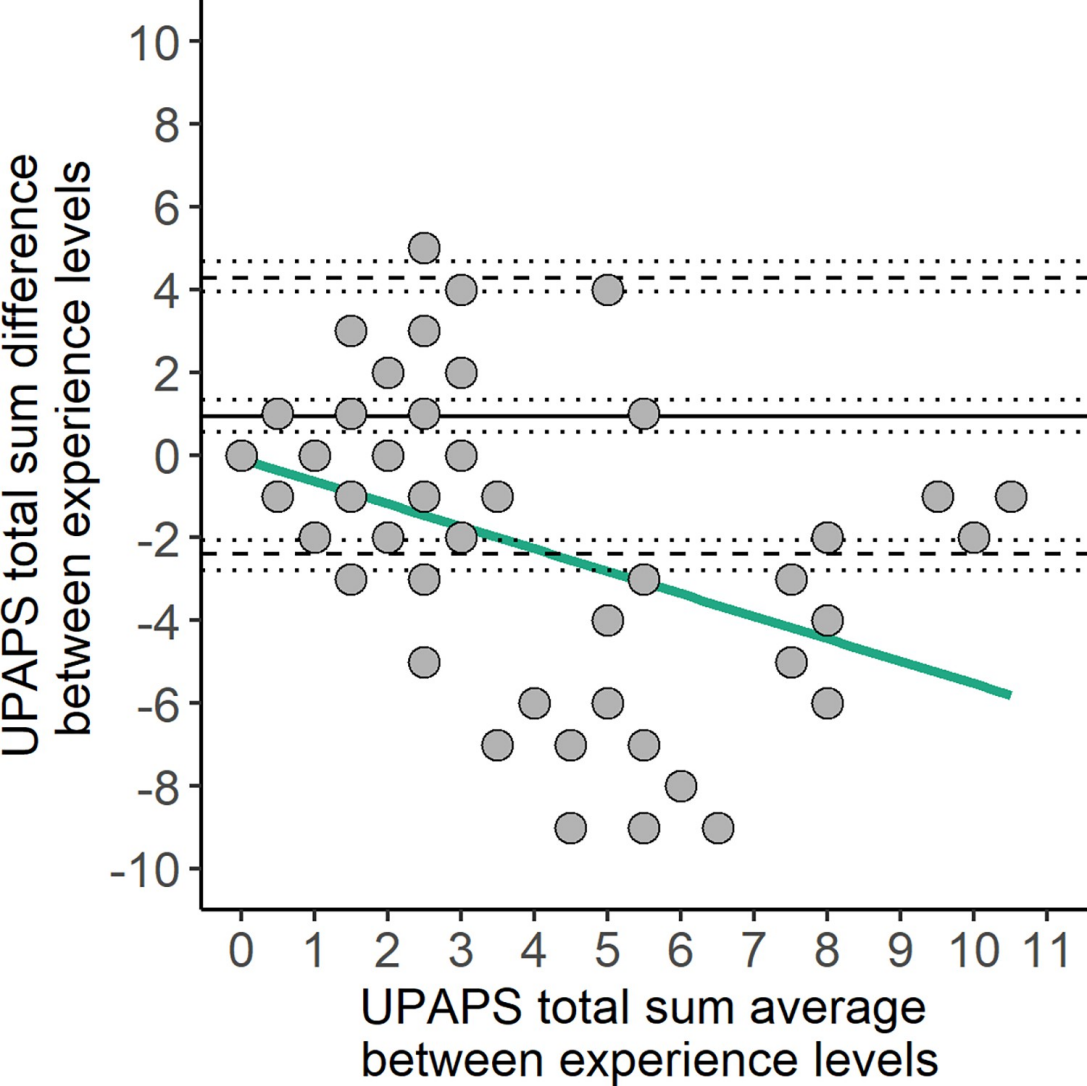
Bland-Altman plot of UPAPS total sum assigned by Little to no experience vs Some experience observers. Solid lines represent bias, dashed lines represent the lower and upper limit of agreement, dotted lines represent the 95% and 90% confidence intervals for bias and lower and upper limit of agreement, respectively. Green line represents the simple linear regression line. UPAPS: Unesp-Botucatu Pig Composite Acute Pain Scale.

**Fig 3:**
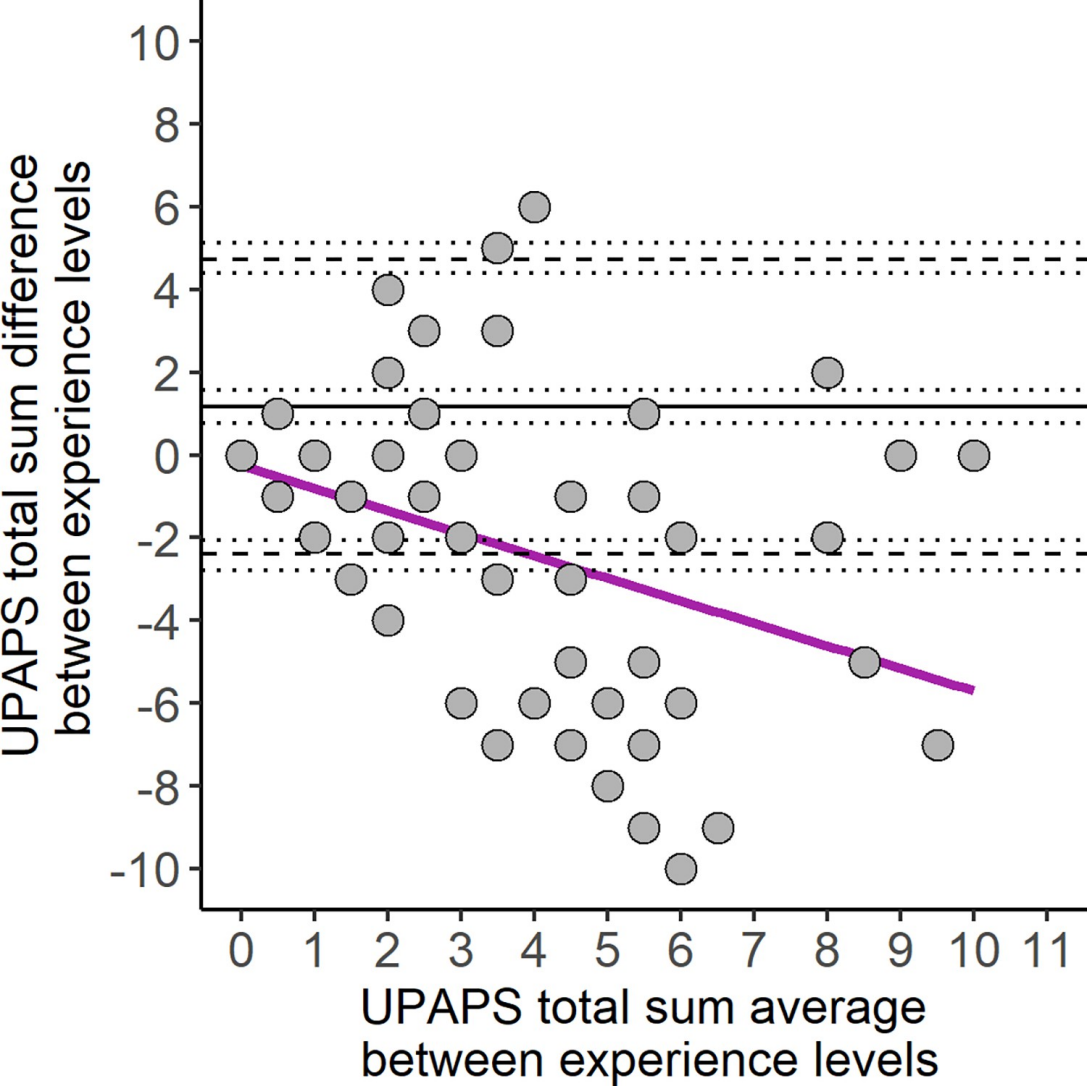
Bland-Altman plot of UPAPS total sum assigned by Little to no experience vs Extensive experience observers. Solid lines represent bias, dashed lines represent the lower and upper limit of agreement, dotted lines represent the 95% and 90% confidence intervals for bias and lower and upper limit of agreement, respectively. Purple line represents the simple linear regression line. UPAPS: Unesp-Botucatu Pig Composite Acute Pain Scale.

**Fig 4:**
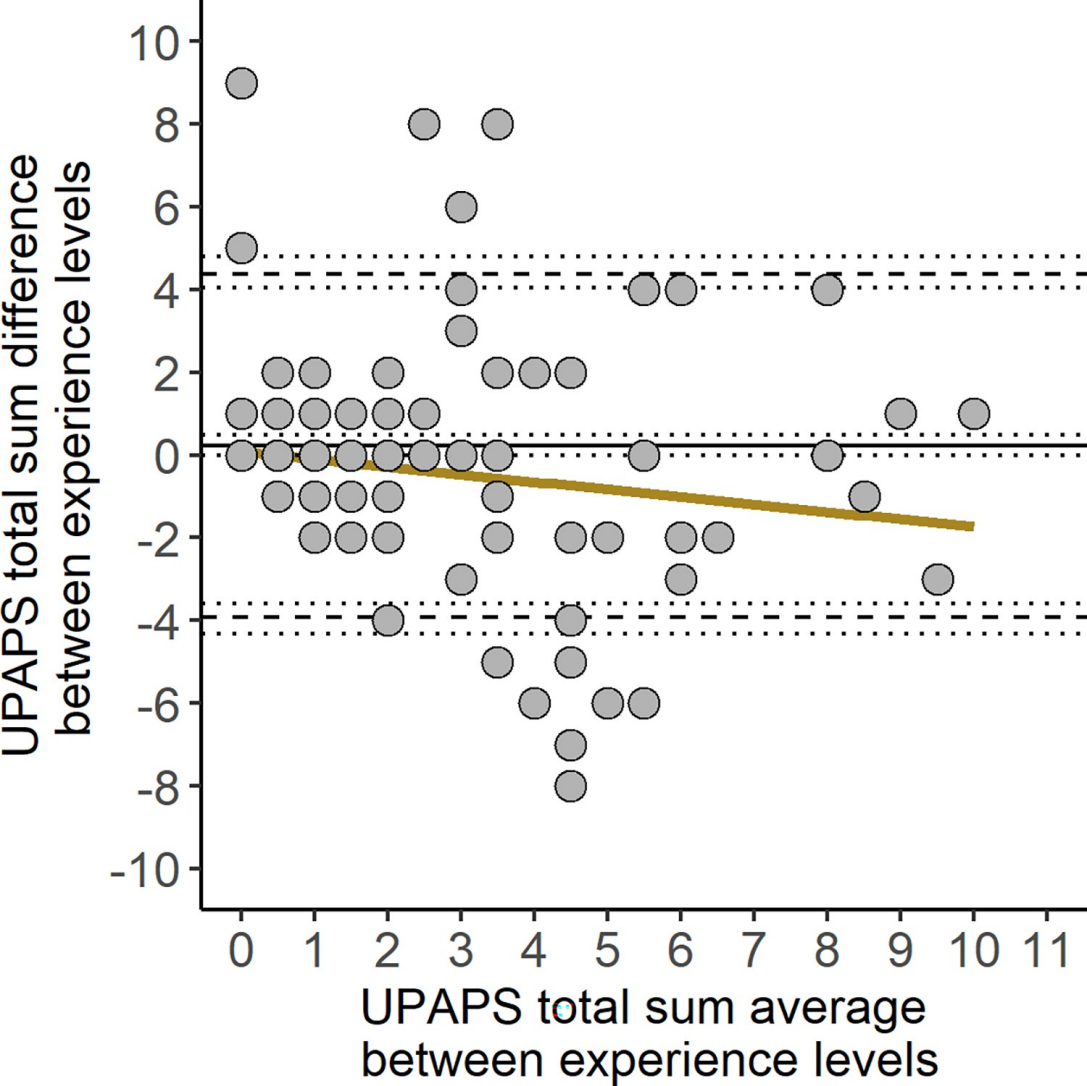
Bland-Altman plot of UPAPS total sum assigned by Some experience vs Extensive experience. Solid lines represent bias, dashed lines represent the lower and upper limit of agreement, dotted lines represent the 95% and 90% confidence intervals for bias and lower and upper limit of agreement, respectively. Yellow line represents the simple linear regression line. UPAPS: Unesp-Botucatu Pig Composite Acute Pain Scale.

Bland-Altman analysis included three agreement assessments: Little to no experience vs Some experience (Fig 2), Little to no experience vs Extensive experience (Fig 3) and Some experience vs Extensive experience (Fig 4). All three had a high bias, combined with an unbalanced limit of agreement between Little to no experience and other experience levels (Table 3). The concordance correlation coefficient was higher in the agreement between Some experience vs Extensive experience (0.72), and lower between Little to no experience vs Extensive experience (0.44). Agreement beyond the limit of agreement ranged from 9.20 to 17.82% in all agreements (Table 3). Only agreements with Little to no experience level showed proportional bias (p < 0.001, S2 Table), and all three agreements had heteroskedasticity (p < 0.001, S2 Table).

**Table 3:** Bland-Altman analyses for the three possible combinations for all experience levels.

Estimates	Experience level groups		
	Little to no experience vs Some experience	Little to no experience vs Extensive experience	Some experience vs Extensive experience
Bias (95% CI)	0.94 (0.55–1.32)	1.17 (0.76–1.57)	0.23 (-0.02–0.47)
Lower LoA (90% CI)	-2.39 (-2.80 - -2.06)	-2.39 (-2.80 - -2.06)	-3.92 (-4.33 - -3.59)
Upper LoA (90% CI)	4.27 (3.94–4.67)	4.72 (4.39–5.13)	4.38 (4.05–4.79)
CCC (95% CI)	0.53 (0.40–0.64)	0.44 (0.31–0.55)	0.72 (0.63–0.78)
***Levels of agreement***			
Perfect (%)	40.23	41.95	42.53
Within LoA (%)	45.98	40.23	48.28
Beyond LoA (%)	13.79	17.82	9.20

CI: Confidence interval; LoA: Limit of agreement; CCC: Concordance correlation coefficient.

### Predictive capacity of experience levels

Extensive experience had the greater AUC of 79.09%, compared to 71.94% and 76.10% from Little to no experience and Some experience, respectively (Table 4). Sensitivity increases along with experience level. Specificity was greater than 91% in all three experience levels. All AUC combinations had no significant (p > 0.05) difference based on DeLong test.

**Table 4:** Experience levels predictive capacity on painful and pain free conditions based on timepoints.

Experience level groups	AUC (95% CI)	Sensitivity (95% CI)	Specificity (95% CI)
**Little to no experience**	71.94 (63.48–80.40)	46.55 (32.76–60.34)	100.00 (100.00–100.00)
**Some experience**	76.10 (67.55–84.65)	60.34 (43.10–72.41)	91.38 (82.76–98.28)
**Extensive experience**	79.09 (70.62–87.56)	63.79 (50.00–75.86)	96.55 (87.93–100.00)

AUC: Area under the receiver operating characteristic curve. CI: Confidence interval; Pain free condition was considered 3 h before castration timepoint, and painful condition was considered immediately after castration timepoint, annotated as ‘0’ and ‘1’ for a binomial response variable, respectively

### UPAPS behaviors modeling

Parameters of the multilevel binomial regression for each UPAPS behavior were presented in S2 Table. Post-hoc analysis showed experience level differences for each UPAPS behavior (S3 Table).

**Fig 5:**
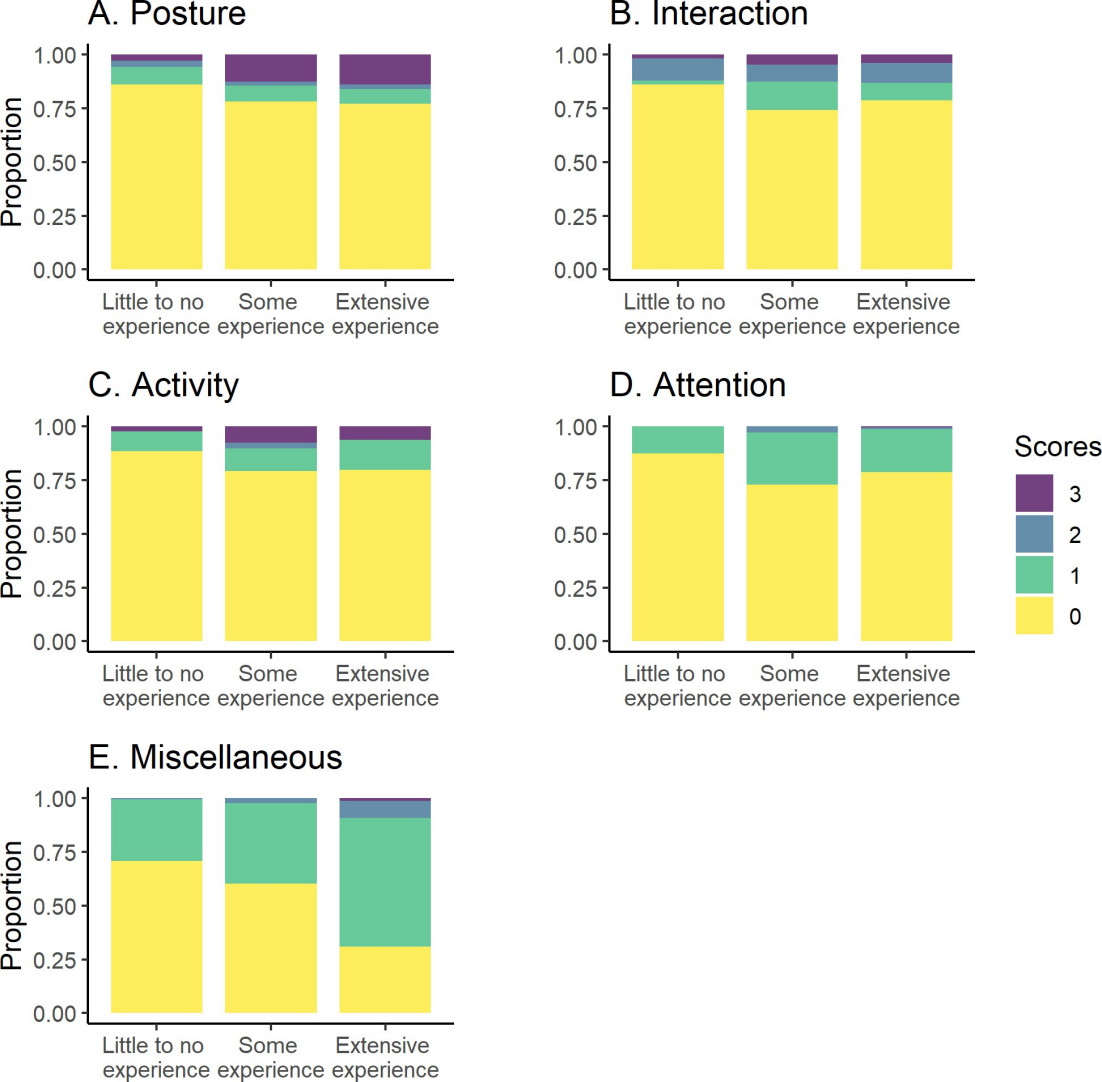
UPAPS scores proportions along experience level groups for UPAPS items. (A) Posture, (B) Interaction, (C) Activity, (D) Attention and (E) Miscellaneous items. UPAPS: Unesp-Botucatu Pig Composite Acute Pain Scale.

When comparing observer experience to specific subitems in the scale, Posture 1 was identified and scored most by observers within the Little to no experience category, followed by observers with Some experience and Extensive experience (Fig 5). Posture 3, Interaction 1, Activity 0, and Head below the line of the spinal column (Miscellaneous behavior) were scored less frequently by observers with Little to no experience compared with the other two levels. Interaction 0 (normal interaction), and an absence of behaviors scored within the attention to the affected area and miscellaneous category was more common for observers with Little to no experience and Some experience compared to Extensive experience. Wags tail continuously and intensely was scored the most by observers within the Extensive experience category.

## Discussion

Advancing pain assessment in swine is an important component for safeguarding animal welfare for livestock used in research and farm settings [8, 26]. The Unesp-Botucatu Pig Composite Acute Pain Scale (UPAPS) is the first validated pain scale used to measure pain in pre-weaned piglets using behavioral assessments scored by observers with extensive species-specific experience [9, 10]. However, to date there are no publications addressing how UPAPS scores would differ from observers with little experience compared with the more experienced ones. Therefore, the objective of this pilot study was to investigate how three different levels of swine experience (no experience, some, extensive) influenced how observers scored castration pain in piglets using UPAPS.

In the current study, three experience levels were evaluated for assessing pain behavior in castrated piglets. During analyzing all six observers combined and comparing paired observers within experience levels, the intraclass correlation coefficient (ICC) demonstrated a “very good” reliability [14]. These results are similar to previous work demonstrating “good” and “very good” ICCs when comparing pain assessment between different observer groups such as cat owners, veterinarians, veterinary students, nurses and cat caregivers using the Feline Grimace Scale [27–29]. It was already expected, as previous studies using UPAPS also demonstrated satisfactory inter-observer reliability [9, 10, 12]. In contrast to the good intra-experience reliability in our study, inter-experience across different experience levels demonstrated only a “moderate” reliability, suggesting pain assessment and diagnosis were influenced by the observers’ experience level with swine. This is likely due to a dilution effect of variance when comparing all six observers simultaneously [30]. The variability when comparing six observers at the same time may be masking the disagreement between observers. In order to more appropriately address this variation, a regression model and Bland-Altman analysis were conducted to further investigate the data.

When comparing pain assessment between experience levels using a regression model and Bland-Altman analysis, Little to no experience observers underscored UPAPS and demonstrated higher bias (greater than one point) [28] and tended to demonstrate greater proportional bias compared to the two other experience levels [21]. This was in contrast to a previous study that observed minimal bias when comparing agreement of the Feline Grimace Scale between cat owners, veterinarians, veterinary students and nurses [28]. In addition to the bias, the limit of agreement (LoA), which was interpreted in relation to the analgesic threshold [31, 32], was unbalanced when comparing Little to no experience observers to Some and Extensive experience observers. Applying this interpretation to our results, the LoA of the little to no experience observers spanned beyond the UPAPS optimal cut-off point of a score equals to 4 [9, 10] and, if used in a clinical or farm settings could make pain diagnosis more difficult, particularly for piglets scoring around the cut-off point. In all comparisons, LoA spanned four, thus indicating that diagnosis around the cut-off point was uncertain between the experience levels. However, when interpreting LoA in agreement with proportional bias, it was thought that LoA should be hyperbolic rather than linear, that was, LoA was wider in the beginning and at the end of the scale points, but narrowed in the middle, where there was a cut-off point.

Deviations in pain assessment scores by Little to no experience observers could also be demonstrated by changes in the ROC curve used to estimate predictive capacity. Although all experience levels had statistically equivalent AUC, sensitivity of Little to no experience observers was lower than the other experience levels and, in a farm setting, would resulted in 13–17% of piglets being misdiagnosed as pain-free (false negative). The results of this study were in contrast with previous work that consistently demonstrated that less experienced observers (i.e. undergraduate students, younger veterinarians and recent graduate students) typically reported higher pain ratings and indicators for analgesic use compared to more experienced professionals [33] in cattle [34–38], dogs [39, 40] and cats [40].

In our study, Little to no experience observers had less than three months of experience working with pigs and these differences may be attributed to the fact that this study was evaluating experience levels with the species and not comparing professional expertise or skill. Less experienced observers like students and early career veterinarians would still likely had extensive experience working with or caring for dogs, cats, horses and/or cattle compared to the limited cultural and logistical opportunities to interact with pigs in the United States, the home country of the observers in the present study. Pigs are not often considered pets or companion animals and opportunities to interact and observe pigs’ behavior are limited. Therefore, comparing the results from this study to previous work might not be appropriate, given non-formal experience with previously assessed species was not addressed.

On the other hand, the results from the current study were similar to work evaluating pain behaviors in rats using the Rat Grimace Scale. Observers with no experience underscored pain behaviors compared to experienced professionals [41], and, as discussed earlier, may be associated with limited casual or informal interaction with rats. Future work should take into account how pain assessment in pigs was influenced beyond experience, and better understand the social and cultural factors that influence our relationships and exposure to the animals we are assessing [1].

Differences between experience levels may also be attributed to scale design. The UPAPS is a questionnaire assessment that needs some level of subjective interpretation by the observer [42]. From an ethological hierarchy [43] standpoint, the scale comprises behaviors more and less complex, which in addition to the scale length, may had reflected in less experienced observers missing behaviors. This was demonstrated by Little to no experience observers tending to score lower on Miscellaneous item, which required attention to and interpretation of multiple behaviors at once. In addition, satisficing is a type of responding bias that occurs when respondents preferably select the first options and are more likely to pay less attention to the latter options [44]. Satisficing would promote underscoring as was found for Little to no experience observers and identifying ways to minimize this bias by simplifying the scales is needed. Recent work from our lab [25, 45] suggests that not all behavioral items are equally weighted and the display of key behaviors such as Wags tail and Posture 1, behaviors that we found differences among experience levels, were important to accurately identify pain states.

Limitations of this study also included some aspects of the experience levels. Because training was provided by an experienced observer, our analysis can only compare if a less experienced observer was able to assess more or less like the experienced observers, however, there is no consensus if naive evaluations are better or worse than those from experienced ones. Thus, the naiveness hypothesis could be further tested in an experiment without training. Another limitation was the low number of observers in each experience level, which can be quite individual. Although we assumed a clear criterion to classify the observers’ experience, in a real-life scenario, the types and intensities of months of experience in the swine industry would differ for each observer. In addition, observers’ general knowledge, age, and personality can contribute to individual variation. This was a pilot study that intended to explore the observers’ experience in the pig acute pain assessment context and can be considered a proposal for research methodology. A larger sample size would be beneficial for estimating differences and addressing diverse backgrounds, cultural and social aspects in future studies.

## Conclusion

Observers with less than three months of experience in the swine industry do not assess pigs’ acute pain response using UPAPS behaviors in an equivalent manner to experienced observers. However, Some experience (3–12 months experience working with pigs) would permit equivalent pain assessment and diagnosis compared to those with extensive experience. The development and refinement of behavioral scales to assess acute pain should be designed and applicable by users with various backgrounds. Beginner-friendly or less complex scale could enhance the implementation of acute pain monitoring in farm and laboratory settings.

## Supporting information

S1 Fig UPAPS score 1 h before castration, immediately post castration and 3 h post castration. (Letters indicate statistical differences (p < 0.05) found in the Bonferroni post-hoc test (a>b>c). UPAPS: Unesp-Botucatu Pig Composite Acute Pain Scale).
(DOCX)

S1 Table UPAPS score modeling using negative binomial multilevel regression. (DOCX)

S2 Table Linear regression model parameters for determining proportional bias and Breusch Pagan test for heteroskedasticity. (DOCX)

S3 Table UPAPS behaviors means ± standard errors of the means across experience levels. (DOCX)

S1 Appendix Dataset used for this study. (CSV)

S2 Appendix Script in R programming language used for the analyses in this study. (DOCX)
