# Supplementary material for: Less experienced observers assess piglet castration-induced acute pain differently than experienced observers: A pilot study
Source: PLoS One. 2024 Sep 4;19(9):e0309684. doi: 10.1371/journal.pone.0309684 (PMC11373819; doi:10.1371/journal.pone.0309684)
Supplement: S1 Fig — (Letters indicate statistical differences (p < 0.05) found in the Bonferroni post-hoc test (a>b>c). UPAPS: Unesp-Botucatu Pig Composite Acute Pain Scale). (DOCX) [file pone.0309684.s001.docx]

**
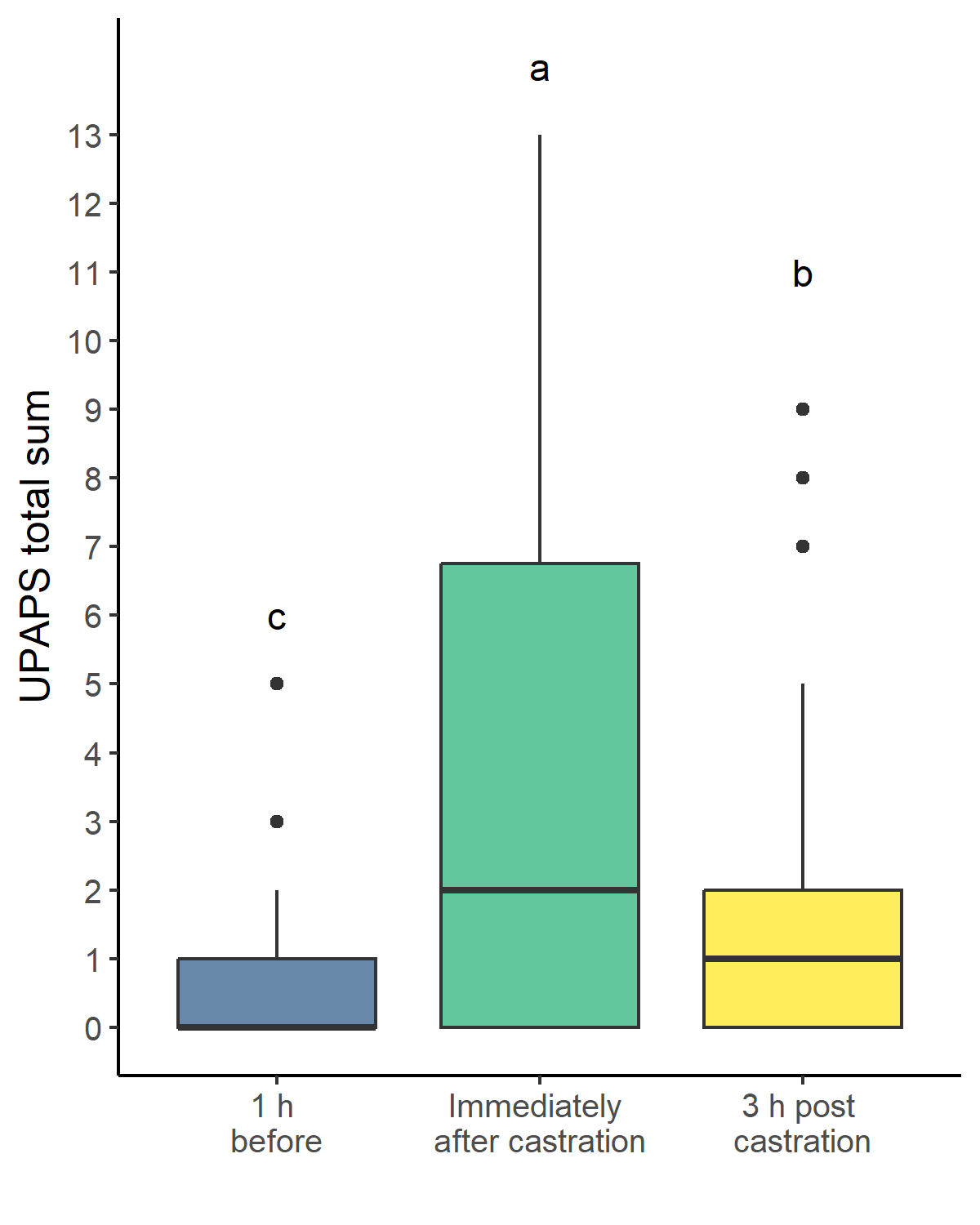
**

**Figure S1**. UPAPS score 1 h before castration, immediately post castration and 3 h post castration. (Letters indicate statistical differences (p < 0.05) found in the Bonferroni post-hoc test (a>b>c). UPAPS: Unesp-Botucatu Pig Composite Acute Pain Scale).
