# Supplementary material for: Less experienced observers assess piglet castration-induced acute pain differently than experienced observers: A pilot study
Source: PLoS One. 2024 Sep 4;19(9):e0309684. doi: 10.1371/journal.pone.0309684 (PMC11373819; doi:10.1371/journal.pone.0309684)
Supplement: S1 Table — (DOCX) [file pone.0309684.s002.docx]

**Table S1.** UPAPS score modeling using negative binomial multilevel regression

| **Fixed effects** | **Estimate** | **SE** | **Z-value** | **p-value** |
| --- | --- | --- | --- | --- |
| *Linear coefficient (α)* | -0.4449 | 0.1937 | -2.2964 | 0.0217 |
|  | | | | |
| *Slope coefficients (β)* | | | | |
| Timepoint 3 h post castration | 0.7715 | 0.1454 | 5.3033 | < 0.0001 |
| Timepoint Immediately post castration | 1.8010 | 0.1347 | 13.3674 | < 0.0001 |
| Experience Little to no | -0.7821 | 0.1237 | -6.3190 | < 0.0001 |
| Experience Some | -0.1562 | 0.1116 | -1.3995 | 0.1616 |
|  |  |  |  |  |
| ***Random effects*** | **Variance** | **SD** | **Observations** |  |
| Piglet:Litter | 0.1619 | 0.4023 | 29 |  |
| Litter | 0.2110 | 0.4593 | 15 |  |

SE: Standard error; SD: Standard deviation
