# Supplementary material for: Less experienced observers assess piglet castration-induced acute pain differently than experienced observers: A pilot study
Source: PLoS One. 2024 Sep 4;19(9):e0309684. doi: 10.1371/journal.pone.0309684 (PMC11373819; doi:10.1371/journal.pone.0309684)
Supplement: S2 Table — (DOCX) [file pone.0309684.s003.docx]

**Table S2.** Linear regression model parameters for determining proportional bias and Breusch Pagan test for heteroskedasticity.

| **Estimates** | **Experience level groups** | | |
| --- | --- | --- | --- |
|  | **Little to no experience vs Some experience** | **Little to no experience vs Extensive experience** | **Some experience vs Extensive experience** |
| ***Linear regression*** |  |  |  |
| Slope coefficient (β) | -0.54 | -0.54 | 0.02 |
| p-value | < 0.0001 | < 0.0001 | 0.6300 |
| ***Breusch Pagan test*** |  |  |  |
| Chi-squared | 134.64 | 154.34 | 93.41 |
| p-value | < 0.0001 | < 0.0001 | < 0.0001 |
