# Supplementary material for: Less experienced observers assess piglet castration-induced acute pain differently than experienced observers: A pilot study
Source: PLoS One. 2024 Sep 4;19(9):e0309684. doi: 10.1371/journal.pone.0309684 (PMC11373819; doi:10.1371/journal.pone.0309684)
Supplement: S3 Table — (DOCX) [file pone.0309684.s004.docx]

**Table S3.** UPAPS behaviors means ± standard errors of the means across experience levels.

| **UPAPS behavior** | **Experience levels** | | |
| --- | --- | --- | --- |
|  | **Little to no** | **Some** | **Extensive** |
| Posture 0 | 0.86 ± 0.03 | 0.78 ± 0.03 | 0.77 ± 0.03 |
| **Posture 1** | **0.08 ± 0.02^a^** | **0.07 ± 0.02^b^** | **0.07 ± 0.02^c^** |
| Posture 2 | 0.03 ± 0.01 | 0.02 ± 0.01 | 0.02 ± 0.01 |
| **Posture 3** | **0.03 ± 0.01^b^** | **0.13 ± 0.03^a^** | **0.14 ± 0.03^a^** |
| **Interaction 0** | **0.86 ± 0.03^a^** | **0.74 ± 0.03^b^** | **0.79 ± 0.03^ab^** |
| **Interaction 1** | **0.02 ± 0.01^a^** | **0.13 ± 0.03^b^** | **0.08 ± 0.02^b^** |
| Interaction 2 | 0.10 ± 0.02 | 0.08 ± 0.02 | 0.09 ± 0.02 |
| Interaction 3 | 0.02 ± 0.01 | 0.05 ± 0.02 | 0.04 ± 0.01 |
| **Activity 0** | **0.89 ± 0.02^a^** | **0.79 ± 0.03^b^** | **0.80 ± 0.03^b^** |
| Activity 1 | 0.09 ± 0.02 | 0.10 ± 0.02 | 0.14 ± 0.03 |
| Activity 2 | 0.00 ± 0.00 | 0.03 ± 0.01 | 0.00 ± 0.00 |
| Activity 3 | 0.02 ± 0.01 | 0.07 ± 0.02 | 0.06 ± 0.02 |
| **Attention 0** | **0.87 ± 0.03^a^** | **0.73 ± 0.03^b^** | **0.79 ± 0.03^ab^** |
| Attention: Elevates pelvic limb | 0.03 ± 0.01 | 0.04 ± 0.01 | 0.04 ± 0.01 |
| Attention: Rubs or scratches affected area | 0.01 ± 0.01 | 0.04 ± 0.01 | 0.03 ± 0.01 |
| Attention: Runs or walks away | 0.00 ± 0.00 | 0.03 ± 0.01 | 0.01 ± 0.01 |
| **Attention: Sits with difficulty** | **0.09 ± 0.02^b^** | **0.18 ± 0.03^a^** | **0.14 ± 0.03^ab^** |
| **Miscellaneous 0** | **0.71 ± 0.03^a^** | **0.60 ± 0.04^a^** | **0.31 ± 0.04^b^** |
| **Miscellaneous: Wags tail** | **0.23 ± 0.03^b^** | **0.26 ± 0.03^b^** | **0.59 ± 0.04^a^** |
| Miscellaneous: Bites bars or objects | 0.00 ± 0.00 | 0.00 ± 0.00 | 0.02 ± 0.01 |
| **Miscellaneous: Head down** | **0.07 ± 0.02^b^** | **0.15 ± 0.03^a^** | **0.18 ± 0.03^a^** |
| Miscellaneous: Difficulty overcoming | 0.00 ± 0.00 | 0.01 ± 0.01 | 0.01 ± 0.01 |

Bold formatting was used to facilitate identification of statistical differences. Letters (a>b>c) indicate statistical differences in the Bonferroni test. The absence of letters indicates no difference. UPAPS: Unesp-Botucatu Pig Composite Acute Pain Scale.
