# Supplementary material for: Less experienced observers assess piglet castration-induced acute pain differently than experienced observers: A pilot study
Source: PLoS One. 2024 Sep 4;19(9):e0309684. doi: 10.1371/journal.pone.0309684 (PMC11373819; doi:10.1371/journal.pone.0309684)
Supplement: S2 Appendix — (DOCX) [file pone.0309684.s006.docx]

#####(0) Loading packages####

pacotes=c(

"dplyr","ggplot2", 'irr', "lme4", "glmmTMB", "lsmeans", "multcomp",

'SimplyAgree','blandr','BlandAltmanLeh', 'gridExtra', "fastDummies",

'pROC'

)

if(sum(as.numeric(!pacotes %in% installed.packages()))!= 0){

instalador=pacotes[!pacotes %in%installed.packages()]

for(i in 1:length(instalador)){

install.packages(instalador,dependencies=T)

break()}

sapply(pacotes,require,character=T)

} else {

sapply(pacotes, require,character=T)

}

set.seed(306)

#####(1) Observers reliability#####

#----Importing data----------------

rm(list=ls())

df_exp = read.csv2("Appendix file S1.csv",h=T,d=",",na.strings=".")

df_select = df_exp[,c("UPAPS")]

#----ICC overall realibility-------

ICC=round(

icc(matrix(c(

df_select[df_exp$Evaluator=="BHR"],

df_select[df_exp$Evaluator=="JF"],

df_select[df_exp$Evaluator=="JS"],

df_select[df_exp$Evaluator=="MLS"],

df_select[df_exp$Evaluator=="MPG"],

df_select[df_exp$Evaluator=="SA"]),nrow=87,ncol=6),model="twoway",unit="average",

type="consistency",conf.level=0.95)$value,dig=2)

ICC_CI_Lower=round(

icc(matrix(c(

df_select[df_exp$Evaluator=="BHR"],

df_select[df_exp$Evaluator=="JF"],

df_select[df_exp$Evaluator=="JS"],

df_select[df_exp$Evaluator=="MLS"],

df_select[df_exp$Evaluator=="MPG"],

df_select[df_exp$Evaluator=="SA"]),nrow=87,ncol=6),model="twoway",unit="average",

type="consistency",conf.level=0.95)$lbound,dig=2)

ICC_CI_Upper=round(

icc(matrix(c(

df_select[df_exp$Evaluator=="BHR"],

df_select[df_exp$Evaluator=="JF"],

df_select[df_exp$Evaluator=="JS"],

df_select[df_exp$Evaluator=="MLS"],

df_select[df_exp$Evaluator=="MPG"],

df_select[df_exp$Evaluator=="SA"]),nrow=87,ncol=6),model="twoway",unit="average",

type="consistency",conf.level=0.95)$ubound,dig=2)

ICC_Pvalue=round(

icc(matrix(c(

df_select[df_exp$Evaluator=="BHR"],

df_select[df_exp$Evaluator=="JF"],

df_select[df_exp$Evaluator=="JS"],

df_select[df_exp$Evaluator=="MLS"],

df_select[df_exp$Evaluator=="MPG"],

df_select[df_exp$Evaluator=="SA"]),nrow=87,ncol=6),model="twoway",unit="average",

type="consistency",conf.level=0.95)$p.value,dig=400)

col0=c(colnames(df_select))

row0=c("ICC",

"ICC_CI_Lower",

"ICC_CI_Upper",

"ICC_Pvalue")

table=matrix(cbind(ICC,

ICC_CI_Lower,

ICC_CI_Upper,

ICC_Pvalue),

nrow=length(col0),ncol=length(row0),dimnames=list(col0,row0))

#----ICC inter group---------------

ICC=round(

icc(matrix(c(

df_select[df_exp$Experience=="Extensive"],

df_select[df_exp$Experience=="Some"],

df_select[df_exp$Experience=="Little"]),nrow=174,ncol=3),model="twoway",unit="average",

type="consistency",conf.level=0.95)$value,dig=2)

ICC_CI_Lower=round(

icc(matrix(c(

df_select[df_exp$Experience=="Extensive"],

df_select[df_exp$Experience=="Some"],

df_select[df_exp$Experience=="Little"]),nrow=174,ncol=3),model="twoway",unit="average",

type="consistency",conf.level=0.95)$lbound,dig=2)

ICC_CI_Upper=round(

icc(matrix(c(

df_select[df_exp$Experience=="Extensive"],

df_select[df_exp$Experience=="Some"],

df_select[df_exp$Experience=="Little"]),nrow=174,ncol=3),model="twoway",unit="average",

type="consistency",conf.level=0.95)$ubound,dig=2)

ICC_Pvalue=round(

icc(matrix(c(

df_select[df_exp$Experience=="Extensive"],

df_select[df_exp$Experience=="Some"],

df_select[df_exp$Experience=="Little"]),nrow=174,ncol=3),model="twoway",unit="average",

type="consistency",conf.level=0.95)$p.value,dig=400)

#----ICC intra group---------------

ICC=round(

icc(matrix(c(

df_select[df_exp$Evaluator=="SA"],

df_select[df_exp$Evaluator=="JF"]),nrow=87,ncol=2),model="twoway",unit="average",

type="consistency",conf.level=0.95)$value,dig=2)

ICC_CI_Lower=round(

icc(matrix(c(

df_select[df_exp$Evaluator=="SA"],

df_select[df_exp$Evaluator=="JF"]),nrow=87,ncol=2),model="twoway",unit="average",

type="consistency",conf.level=0.95)$lbound,dig=2)

ICC_CI_Upper=round(

icc(matrix(c(

df_select[df_exp$Evaluator=="SA"],

df_select[df_exp$Evaluator=="JF"]),nrow=87,ncol=2),model="twoway",unit="average",

type="consistency",conf.level=0.95)$ubound,dig=2)

ICC_Pvalue=round(

icc(matrix(c(

df_select[df_exp$Evaluator=="SA"],

df_select[df_exp$Evaluator=="JF"]),nrow=87,ncol=2),model="twoway",unit="average",

type="consistency",conf.level=0.95)$p.value,dig=400)

#####(2) Bland-Altman analysis#####

#-----Importing data-----

rm(list=ls())

df_exp = read.csv2("exp_ba.csv",h=T,d=",",na.strings=".")

#-----Analysis-----

# Little vs Some

ba_little_some = agree_reps(y = "upaps_little",

x = "upaps_some",

id = "id",

data = df_exp,

agree.level = .95,

prop_bias=TRUE);ba_little_some

# Little vs Extensive# LTRUEittle vs Extensive

ba_little_ext =agree_reps(y = "upaps_little",

x = "upaps_extensive",

id = "id",

data = df_exp,

agree.level = .95,

prop_bias=TRUE);ba_little_ext

# Some vs Extensive

ba_some_ext =agree_reps(y = "upaps_some",

x = "upaps_extensive",

id = "id",

data = df_exp,

agree.level = .95);ba_some_ext

#-----Levels of agreement-----

#Little vs some

(sum(df_exp$diff_1 == 0)/length(df_exp$diff_1)) * 100

(sum(df_exp$diff_1 <= 4.27 & df_exp$diff_1 >= -2.39 & df_exp$diff_1 != 0)/length(df_exp$diff_1)) * 100

(sum(df_exp$diff_1 > 4.27 | df_exp$diff_1 < -2.39)/length(df_exp$diff_1)) * 100

#Little vs extensive

(sum(df_exp$diff_2 == 0)/length(df_exp$diff_2)) * 100

(sum(df_exp$diff_2 <= 4.72 & df_exp$diff_2 >= -2.39 & df_exp$diff_2 != 0)/length(df_exp$diff_2)) * 100

(sum(df_exp$diff_2 > 4.72 | df_exp$diff_2 < -2.39)/length(df_exp$diff_2)) * 100

#Some vs extensive

(sum(df_exp$diff_3 == 0)/length(df_exp$diff_3)) * 100

(sum(df_exp$diff_3 <= 4.38 & df_exp$diff_3 >= -3.92 & df_exp$diff_3 != 0)/length(df_exp$diff_3)) * 100

(sum(df_exp$diff_3 > 4.38 | df_exp$diff_3 < -3.92)/length(df_exp$diff_3)) * 100

#-----Proportional bias-----

summary(lm(diff_1~avg_1,df_exp))

summary(lm(diff_2~avg_2,df_exp))

summary(lm(diff_3~avg_3,df_exp))

#-----Heteroskedasticity----

olsrr::ols_test_breusch_pagan(lm(diff_1~avg_1,df_exp))

olsrr::ols_test_breusch_pagan(lm(diff_2~avg_2,df_exp))

olsrr::ols_test_breusch_pagan(lm(diff_3~avg_3,df_exp))

#####(3) Negative binomial modeling#####

#-----Importing data-----

rm(list=ls())

df=read.csv2(

"experience.csv"

,h=T

,d=","

,na.strings='.')

#-----Modeling-----

mod=glmer.nb(UPAPS~Timepoint*Experience*Gender+(1|Litter/Piglet),data=df,

control=glmerControl(optimizer="bobyqa",optCtrl=list(maxfun=100000)))

mod2=glmer.nb(UPAPS~Timepoint+Experience+(1|Litter/Piglet),data=df,

control=glmerControl(optimizer="bobyqa",optCtrl=list(maxfun=100000)))

aa = summary(mod2)

BIC(mod, mod2)

#-----Post hoc-----

ph_experience = lsmeans(mod2,pairwise~Experience,p.adjust="bonferroni", type = "response")

cld(ph_experience ,Letters="abcde",alpha=.05)

#####()

#####(4) ROC analysis#####

#-----Importing data-----

rm(list=ls())

df_exp = read.csv2("experience.csv",h=T,d=",",na.strings=".")

#-----ROC curve and estimates-----

df_temp = df_exp[df_exp$Timepoint == "1hbefore" | df_exp$Timepoint == "PostCastration",]

roc_little = roc(Timepoint~UPAPS, df_temp[df_temp$Experience == "Little",], plot=F,

algorithm=2, smooth=F, boot.n=1001, boot.stratified=T, ci.auc=T, auc=T)

roc_cicoords_little = ci.coords(roc_little,x="best",

input=c("threshold", "specificity", "sensitivity"),

ret=c("threshold", "specificity", "sensitivity"),

best.method="youden",

best.policy = "random",

conf.level=0.95, boot.n=1001,

boot.stratified=TRUE)

roc_some = roc(Timepoint~UPAPS, df_temp[df_temp$Experience == "Some",], plot=F,

algorithm=2, smooth=F, boot.n=1001, boot.stratified=T, ci.auc=T, auc=T)

roc_cicoords_some = ci.coords(roc_some,x="best",

input=c("threshold", "specificity", "sensitivity"),

ret=c("threshold", "specificity", "sensitivity"),

best.method="youden",

best.policy = "random",

conf.level=0.95, boot.n=1001,

boot.stratified=TRUE)

roc_extensive = roc(Timepoint~UPAPS, df_temp[df_temp$Experience == "Extensive",], plot=F,

algorithm=2, smooth=F, boot.n=1001, boot.stratified=T, ci.auc=T, auc=T)

roc_cicoords_extensive = ci.coords(roc_extensive,x="best",

input=c("threshold", "specificity", "sensitivity"),

ret=c("threshold", "specificity", "sensitivity"),

best.method="youden",

best.policy = "random",

conf.level=0.95, boot.n=1001,

boot.stratified=TRUE)

#-----DeLong--------

roc.test(roc_little, roc_some)

roc.test(roc_little, roc_extensive)

roc.test(roc_some, roc_extensive)

#####(5) Behavior modeling#####

#-----Importing data-----

rm(list=ls())

df_exp = read.csv2("experience.csv",h=T,d=",",na.strings=".")

#-----Dummy columns-----

df_dummy = df_exp[,c(3:5, 7:21, 23)]

df_dummy = dummy_columns(df_dummy, select_columns=colnames(df_dummy[,c(6:18)]),

remove_selected_columns=T,

remove_first_dummy=F)

#-----Modeling-----

for (i in c(7:19, 24:31, 36:42)) {

print(colnames(df_dummy[i]))

response = df_dummy[,i]

mod1 = glmer(response~ Experience+(1|Litter/Piglet),data=df_dummy,

family="binomial",

control=glmerControl(optimizer="bobyqa",optCtrl=list(maxfun=100000)))

print(summary(mod1)$coefficients)

ph_experience = lsmeans(mod1,pairwise~Experience,p.adjust="bonferroni", type = "response")

print(cld(ph_experience ,Letters="abcde",alpha=.05)[,c(1, 7)])

}

#-----Post hoc and descriptives-----

descriptive = data.frame (behavior = character(),

experience = character(),

mean = numeric())

std.error <- function(x) sd(x)/sqrt(length(x))

for (i in c(7:19, 24:31, 36:42)){

b_temp = colnames(df_dummy[i])

for (j in (c("Little", "Some", "Extensive"))) {

df_temp = df_dummy[df_dummy$Experience == j,]

e_temp = j

m_temp = paste(round(mean(df_temp[,i]),2), " ± ",

round(std.error(df_temp[,i]),2), sep="")

descriptive = rbind(descriptive, data.frame(behavior=b_temp, experience=e_temp, mean=m_temp))

} }

write.csv2(descriptive, "(4) descriptive.csv", row.names=F)

#####(6) Figures#####

df_exp$Evaluator = factor(df_exp$Evaluator,

labels=c("1",

'2',

'3',

'4',

'5',

'6'),

levels=c("SA", "JF",

"BHR", "JS",

"MLS", "MPG"))

df_exp$Timepoint = factor(df_exp$Timepoint,

labels=c("1 h \nbefore",

"Immediately \nafter castration",

"3 h post \ncastration"),

levels=c("1hbefore",

"PostCastration",

"3hPostCastration"))

df_exp$Experience = factor(df_exp$Experience,

labels=c("Little to no \nexperience",

"Some \nexperience",

"Extensive \nexperience"),

levels=c("Little",

"Some",

"Extensive"))

library(patchwork)

#----1. Observers scores-----------

evaluators = df_exp %>%

ggplot(aes(x=Evaluator, y=UPAPS, fill=Experience))+

geom_boxplot()+

theme_classic()+

scale_y_continuous(n.breaks = 14, limits = c(0,14),

labels=c(0, 1, 2, 3, 4, 5,

6, 7, 8, 9, 10, 11,

12, 13),

breaks=c(0, 1, 2, 3, 4, 5,

6, 7, 8, 9, 10, 11,

12, 13))+

labs(y="UPAPS total sum", x="Observers", tag="A")+

theme(panel.background = element_blank(),

text = element_text(size=14),

plot.margin = unit(c(1,0,0,1),"cm"))+

scale_fill_viridis_d(alpha=.75, end=0.7);evaluators

score_plot = df_exp %>%

ggplot(aes(x=Experience, y=UPAPS, fill=Experience))+

geom_boxplot(width=0.5)+

theme_classic()+

scale_y_continuous(n.breaks = 14, limits = c(0,14),

labels=c(0, 1, 2, 3, 4, 5,

6, 7, 8, 9, 10, 11,

12, 13),

breaks=c(0, 1, 2, 3, 4, 5,

6, 7, 8, 9, 10, 11,

12, 13))+

labs(y="UPAPS total sum", x="", tag="B")+

theme(panel.background = element_blank(),

legend.position= "none",

text = element_text(size=14),

plot.margin = unit(c(0,0,1,1),"cm"))+

annotate("text", x=1, y=11, label="b")+

annotate("text", x=2, y=12, label="a")+

annotate("text", x=3, y=14, label="a")+

scale_fill_viridis_d(alpha=.75, end=.7);score_plot

tiff("Figure 1.tiff",width=5.2,height=6.5,units='in',res=300,

compression = "lzw",

family="sans")

#png("(1) Score by observer and experience.png", width = 5, height = 6.5, units = 'in', res=300, pointsize=12)

evaluators + score_plot + plot_layout(guides="collect", ncol=1)

dev.off()

#----3. Bland Altman plots-----

####Little vs some

df_exp$avg_1 = rowMeans(df_exp[,c(2,3)])

df_exp$diff_1 = df_exp$upaps_little - df_exp$upaps_some

mean_diff_1=mean(df_exp$diff_1)

lower_1=mean_diff_1 - 1.96*sd(df_exp$diff_1)

upper_1=mean_diff_1 + 1.96*sd(df_exp$diff_1)

ba_little_some$loa

a = ggplot(df_exp, aes(x = avg_1, y = diff_1)) +

geom_smooth(method="lm",formula=y~x,se=F,show.legend=F,linewidth=1.2,alpha=.18,color='#22A884FF')+

geom_hline(yintercept = ba_little_some$loa[[1,1]]) +

geom_hline(yintercept = ba_little_some$loa[[1,2]],linetype="dotted") +

geom_hline(yintercept = ba_little_some$loa[[1,3]],linetype="dotted") +

geom_hline(yintercept = ba_little_some$loa[[2,1]], color = "black", linetype="dashed") +

geom_hline(yintercept = ba_little_some$loa[[2,2]],linetype="dotted") +

geom_hline(yintercept = ba_little_some$loa[[2,3]],linetype="dotted") +

geom_hline(yintercept = ba_little_some$loa[[3,1]], color = "black", linetype="dashed") +

geom_hline(yintercept = ba_little_some$loa[[3,2]],linetype="dotted") +

geom_hline(yintercept = ba_little_some$loa[[3,3]],linetype="dotted") +

geom_point(size=3.4,fill='gray70',color='black',shape=21,alpha=6) +

#ggtitle("Both genders evaluators") +

ylab("UPAPS total sum difference\n between experience levels") +

xlab("UPAPS total sum average\n between experience levels")+

#labs(title="A. Little to no experience \nvs Some experience")+

scale_y_continuous(n.breaks=14,limits = c(-10,10))+

scale_x_continuous(n.breaks=13,limits = c(0,11))+

theme_classic()+theme(axis.text=element_text(size=12),

axis.title=element_text(size=12),

legend.text=element_text(size=12),

legend.title = element_text(size=12),

panel.grid.minor = element_blank(),

panel.grid.major = element_blank(),

plot.margin = unit(c(1,1,1,1),"cm"),

panel.background = element_blank()) +

annotate("text",x=15,y=12,size=4.8,hjust='right',

label="Bias: 0.94 (CI: 0.55 to 1.32)\nLower LoA: -2.10 (CI: -2.54 to -1.71)\nUpper LoA: 3.97 (CI: 3.58 to 4.41)\nCCC: 0.53 (CI: 0.40 to 0.64)"); a

tiff("Figure 2.tiff",width=4.5,height=4.5,units='in',res=300,

compression = "lzw",

family="sans")

a

dev.off()

####Little vs extensive

df_exp$avg_2 = rowMeans(df_exp[,c(2,4)])

df_exp$diff_2 = df_exp$upaps_little - df_exp$upaps_extensive

mean_diff_2=mean(df_exp$diff_2)

lower_2=mean_diff_2 - 1.96*sd(df_exp$diff_2)

upper_2=mean_diff_2 + 1.96*sd(df_exp$diff_2)

b = ggplot(df_exp, aes(x = avg_2, y = diff_2)) +

geom_smooth(method="lm",formula=y~x,se=F,show.legend=F,linewidth=1.2,alpha=.18,color='#a622a8')+

geom_hline(yintercept = ba_little_ext$loa[[1,1]]) +

geom_hline(yintercept = ba_little_ext$loa[[1,2]],linetype="dotted") +

geom_hline(yintercept = ba_little_ext$loa[[1,3]],linetype="dotted") +

geom_hline(yintercept = ba_little_ext$loa[[2,1]], color = "black", linetype="dashed") +

geom_hline(yintercept = ba_little_ext$loa[[2,2]],linetype="dotted") +

geom_hline(yintercept = ba_little_ext$loa[[2,3]],linetype="dotted") +

geom_hline(yintercept = ba_little_ext$loa[[3,1]], color = "black", linetype="dashed") +

geom_hline(yintercept = ba_little_ext$loa[[3,2]],linetype="dotted") +

geom_hline(yintercept = ba_little_ext$loa[[3,3]],linetype="dotted") +

geom_point(size=3.4,fill='gray70',color='black',shape=21,alpha=6) +

#ggtitle("Both genders evaluators") +

ylab("UPAPS total sum difference\n between experience levels") +

xlab("UPAPS total sum average\n between experience levels")+

#labs(title="B. Little to no experience \nvs Extensive experience")+

scale_y_continuous(n.breaks=14,limits = c(-10,10))+

scale_x_continuous(n.breaks=13,limits = c(0,11))+

theme_classic()+theme(axis.text=element_text(size=12),

axis.title=element_text(size=12),

legend.text=element_text(size=12),

legend.title = element_text(size=12),

panel.grid.minor = element_blank(),

panel.grid.major = element_blank(),

plot.margin = unit(c(1,1,1,1),"cm"),

panel.background = element_blank()) +

annotate("text",x=15,y=12,size=4.8,hjust='right',

label="Bias: 1.17 (CI: 0.76 to 1.57)\nLower LoA: -2.03 (CI: -2.49 to -1.62)\nUpper LoA: 4.36 (CI: 3.95 to 4.83)\nCCC: 0.44 (CI: 0.31 to 0.55)"); b

tiff("Figure 3.tiff",width=4.5,height=4.5,units='in',res=300,

compression = "lzw",

family="sans")

b

dev.off()

####Some vs extensive

df_exp$avg_3 = rowMeans(df_exp[,c(3,4)])

df_exp$diff_3 = df_exp$upaps_some - df_exp$upaps_extensive

mean_diff_3=mean(df_exp$diff_3)

lower_3=mean_diff_3 - 1.96*sd(df_exp$diff_3)

upper_3=mean_diff_3 + 1.96*sd(df_exp$diff_3)

c = ggplot(df_exp, aes(x = avg_2, y = diff_3)) +

geom_smooth(method="lm",formula=y~x,se=F,show.legend=F,linewidth=1.2,alpha=.18,color='#a88722')+

geom_hline(yintercept = ba_some_ext$loa[[1,1]]) +

geom_hline(yintercept = ba_some_ext$loa[[1,2]],linetype="dotted") +

geom_hline(yintercept = ba_some_ext$loa[[1,3]],linetype="dotted") +

geom_hline(yintercept = ba_some_ext$loa[[2,1]], color = "black", linetype="dashed") +

geom_hline(yintercept = ba_some_ext$loa[[2,2]],linetype="dotted") +

geom_hline(yintercept = ba_some_ext$loa[[2,3]],linetype="dotted") +

geom_hline(yintercept = ba_some_ext$loa[[3,1]], color = "black", linetype="dashed") +

geom_hline(yintercept = ba_some_ext$loa[[3,2]],linetype="dotted") +

geom_hline(yintercept = ba_some_ext$loa[[3,3]],linetype="dotted") +

geom_point(size=3.4,fill='gray70',color='black',shape=21,alpha=6) +

#ggtitle("Both genders evaluators") +

ylab("UPAPS total sum difference\n between experience levels") +

xlab("UPAPS total sum average\n between experience levels")+

#labs(title="C. Some experience \nvs Extensive experience")+

scale_y_continuous(n.breaks=14,limits = c(-10,10))+

scale_x_continuous(n.breaks=13,limits = c(0,11))+

theme_classic()+theme(axis.text=element_text(size=12),

axis.title=element_text(size=12),

legend.text=element_text(size=12),

legend.title = element_text(size=12),

panel.grid.minor = element_blank(),

panel.grid.major = element_blank(),

plot.margin = unit(c(1,1,1,1),"cm"),

panel.background = element_blank()) +

annotate("text",x=15,y=12,size=4.8,hjust='right',

label="Bias: 0.23 (CI: -0.02 to 0.47)\nLower LoA: -2.48 (CI: -2.80 to -2.22)\nUpper LoA: 2.94 (CI: 2.68 to 3.25)\nCCC: 0.72 (CI: 0.63 to 0.78)"); c

tiff("Figure 4.tiff",width=4.5,height=4.5,units='in',res=300,

compression = "lzw",

family="sans")

c

dev.off()

png("(3) Bland-Altman plots.png", width = 9, height = 9, units = 'in', res=300, pointsize=12)

grid.arrange(a, b, c, layout_matrix = layout_matrix)

dev.off()

#----4. Scores proportion------

df_proportion_posture <- df_exp %>%

group_by(Experience, Posture) %>%

summarise(Count = n()) %>%

group_by(Experience) %>%

mutate(Proporcao = Count / sum(Count))

df_proportion_posture$Posture = factor(df_proportion_posture$Posture,

levels=c(3, 2, 1, 0))

a = df_proportion_posture %>%

ggplot(aes(x=Experience, y=Proporcao, fill=Posture))+

geom_bar(stat="identity", width=0.8)+

labs(title="A. Posture", fill="Scores", y="Proportion", x="")+

scale_fill_viridis_d(alpha=0.75)+

#scale_fill_manual(values=c("#56b1f7", "#3e81b7", "#28547a", "#132b43"))+

theme_classic()+

theme(text=element_text(size=12),

plot.margin = unit(c(1,0, 0, 1),"cm"));a

df_proportion_interac <- df_exp %>%

group_by(Experience, Interac) %>%

summarise(Count = n()) %>%

group_by(Experience) %>%

mutate(Proporcao = Count / sum(Count))

df_proportion_interac$Interac = factor(df_proportion_interac$Interac,

levels=c(3, 2, 1, 0))

b = df_proportion_interac %>%

ggplot(aes(x=Experience, y=Proporcao, fill=Interac))+

geom_bar(stat="identity", width=0.8)+

theme_classic()+

labs(title="B. Interaction", y="Proportion", x="")+

scale_fill_viridis_d(alpha=0.75)+

#scale_fill_manual(values=c("#56b1f7", "#3e81b7", "#28547a", "#132b43"))+

theme_classic()+

theme(legend.position="none",

text=element_text(size=12),

plot.margin = unit(c(1, 1, 0, 0),"cm"));b

df_proportion_activity <- df_exp %>%

group_by(Experience, Activity) %>%

summarise(Count = n()) %>%

group_by(Experience) %>%

mutate(Proporcao = Count / sum(Count))

df_proportion_activity$Activity = factor(df_proportion_activity$Activity,

levels=c(3, 2, 1, 0))

c = df_proportion_activity %>%

ggplot(aes(x=Experience, y=Proporcao, fill=Activity))+

geom_bar(stat="identity", width=0.8)+

theme_classic()+

labs(title="C. Activity", y="Proportion", x="")+

scale_fill_viridis_d(alpha=0.75)+

#scale_fill_manual(values=c("#56b1f7", "#3e81b7", "#28547a", "#132b43"))+

theme_classic()+

theme(legend.position="none",

text=element_text(size=12),

plot.margin = unit(c(0,0, 0, 1),"cm"));c

df_proportion_attention<- df_exp %>%

group_by(Experience, Attention) %>%

summarise(Count = n()) %>%

group_by(Experience) %>%

mutate(Proporcao = Count / sum(Count))

df_proportion_attention$Attention = factor(df_proportion_attention$Attention,

levels=c(3, 2, 1, 0))

d = df_proportion_attention %>%

ggplot(aes(x=Experience, y=Proporcao, fill=Attention))+

geom_bar(stat="identity", width=0.8)+

theme_classic()+

labs(title="D. Attention", y="Proportion", x="")+

scale_fill_viridis_d(alpha=0.75)+

#scale_fill_manual(values=c("#56b1f7", "#3e81b7", "#28547a", "#132b43"))+

theme_classic()+

theme(legend.position="none",

text=element_text(size=12),

plot.margin = unit(c(0,0, 0, 1),"cm"));d

df_proportion_miscellaneous <- df_exp %>%

group_by(Experience, Miscellaneous) %>%

summarise(Count = n()) %>%

group_by(Experience) %>%

mutate(Proporcao = Count / sum(Count))

df_proportion_miscellaneous$Miscellaneous = factor(df_proportion_miscellaneous$Miscellaneous,

levels=c(3, 2, 1, 0))

e = df_proportion_miscellaneous %>%

ggplot(aes(x=Experience, y=Proporcao, fill=Miscellaneous))+

geom_bar(stat="identity", width=0.8)+

theme_classic()+

labs(title="E. Miscellaneous", y="Proportion", x="")+

scale_fill_viridis_d(alpha=0.75)+

#scale_fill_manual(values=c("#56b1f7", "#3e81b7", "#28547a", "#132b43"))+

theme_classic()+

theme(legend.position="none",

text=element_text(size=12),

plot.margin = unit(c(0, 0, 1, 1),"cm"));e

df_exp$Scores = factor(df_exp$Posture, labels=c(0, 1, 2, 3),

levels=c(0, 1, 2, 3))

library(patchwork)

tiff("Figure 5.tiff",width=7.5,height=7.5,units='in',res=300,

compression = "lzw",

family="sans")

#png("(4) Score proportions option 2.png", width = 6.5, height = 7.5, units = 'in', res=300, pointsize=12)

a+b+c+d+e+plot_layout(guides="collect", ncol=2)

dev.off()

#----Suppl Fig 1. Timepoint scores----

png("(1) UPAPS score timepoints.png", width = 4, height = 5, units = 'in', res=300, pointsize=12)

df_exp %>%

ggplot(aes(x=Timepoint, y=UPAPS, fill=Timepoint))+

geom_boxplot()+

theme_classic()+

scale_y_continuous(n.breaks = 14, limits = c(0,14),

labels=c(0, 1, 2, 3, 4, 5,

6, 7, 8, 9, 10, 11,

12, 13),

breaks=c(0, 1, 2, 3, 4, 5,

6, 7, 8, 9, 10, 11,

12, 13))+

labs(y="UPAPS total sum", x="")+

theme(panel.background = element_blank(),

legend.position= "none",

text=element_text(size=12))+

annotate("text", x=1, y=6, label="c")+

annotate("text", x=2, y=14, label="a")+

annotate("text", x=3, y=11, label="b")+

scale_fill_viridis_d(alpha=.75, begin=.3)

dev.off()
